# The Vaginal Microbial Signatures of Preterm Birth Delivery in Indian Women

**DOI:** 10.3389/fcimb.2021.622474

**Published:** 2021-05-13

**Authors:** Shakti Kumar, Naina Kumari, Daizee Talukdar, Akansha Kothidar, Mousumi Sarkar, Ojasvi Mehta, Pallavi Kshetrapal, Nitya Wadhwa, Ramachandran Thiruvengadam, Bapu Koundinya Desiraju, G. Balakrish Nair, Shinjini Bhatnagar, Souvik Mukherjee, Bhabatosh Das, GARBH-Ini Study Group, Shinjini Bhatnagar

**Affiliations:** ^1^ Molecular Genetics Laboratory, Translational Health Science and Technology Institute, National Capital Region (NCR) Biotech Science Cluster, Faridabad, India; ^2^ National Institute of Biomedical Genomics, Kalyani, India; ^3^ Pediatric Biology Center, Translational Health Science and Technology Institute, NCR Biotech Science Cluster, Faridabad, India

**Keywords:** vaginal microbiota, microbial ecology, *Lactobacillus*, preterm birth, 16S rRNA gene sequencing

## Abstract

**Background:**

The incidence of preterm birth (PTB) in India is around 13%. Specific bacterial communities or individual taxon living in the vaginal milieu of pregnant women is a potential risk factor for PTB and may play an important role in its pathophysiology. Besides, bacterial taxa associated with PTB vary across populations.

**Objective:**

Conduct a comparative analysis of vaginal microbiome composition and microbial genomic repertoires of women who enrolled in the Interdisciplinary Group for Advanced Research on Birth Outcomes – A DBT India Initiative (GARBH-Ini) pregnancy cohort to identify bacterial taxa associated with term birth (TB) and PTB in Indian women.

**Methods:**

Vaginal swabs were collected during all three trimesters from 38 pregnant Indian women who delivered spontaneous term (n=20) and preterm (n=18) neonates. Paired-end sequencing of V3-V4 region of 16S rRNA gene was performed using the metagenomic DNA isolated from vaginal swabs (n=115). Whole genome sequencing of bacterial species associated with birth outcomes was carried out by shotgun method. *Lactobacillus* species were grown anaerobically in the De Man, Rogosa and Sharpe (MRS) agar culture medium for isolation of genomic DNA and whole genome sequencing.

**Results:**

Vaginal microbiome of both term and preterm samples reveals similar alpha diversity indices. However, significantly higher abundance of *Lactobacillus iners* (p-value _All_Trimesters_<0.02), *Megasphaera* sp (p-value_1st_Trimester <_0.05), *Gardnerella vaginalis* (p-value_2nd_Trimester_= 0.01) and *Sneathia sanguinegens* (p-value_2nd_Trimester <_0.0001) were identified in preterm samples whereas higher abundance of *L. gasseri* (p-value_3rd_Trimester_ =0.010) was observed in term samples by Wilcoxon rank-sum test. The relative abundance of *L. iners*, and *Megasphaera* sp. were found to be significantly different over time between term and preterm mothers. Analyses of the representative genomes of *L. crispatus* and *L. gasseri* indicate presence of secretory transcriptional regulator and several ribosomally synthesized antimicrobial peptides correlated with anti-inflammatory condition in the vagina. These findings indicate protective role of *L. crispatus* and *L. gasseri* in reducing the risk of PTB.

**Conclusion:**

Our findings indicate that the dominance of specific *Lactobacillus* species and few other facultative anaerobes are associated with birth outcomes.

## Introduction

Preterm birth (PTB), defined as birth before 37 completed weeks of gestation, is a major public health problem across the globe. It is one of the leading causes of neonatal mortality and morbidity in developed and developing countries (WHO, 2018). In India, out of 27 million babies born every year, 3.6 million babies are born prematurely (WHO, 2018; Sharma et al., 2018). It accounts for an estimated 40% of neonatal deaths worldwide and affects about 1 in 10 pregnancies every year (WHO, 2018). The consequences of PTB continue from early childhood into adolescence and adulthood (Marret et al., 2007; Wolke et al., 2014). Infants born prematurely also have higher rates of respiratory distress syndrome, cardiovascular disorders, neuro developmental disabilities and learning difficulties as compared to those born at term (TB) (Butler and Behrman, 2007). The underlying etiology that induces PTB may also affect maternal health. Multiple lines of evidence support a role of the vaginal microbial communities in the pathophysiology of PTB delivery (Romero et al., 2014; Klebanoff and Brotman, 2018). Less diverse vaginal microbiota has long been considered the hallmark of reproductive health and associated with TB outcome (Fettweis et al., 2019). In healthy reproductive-aged women, the vaginal microbiome generally shows a predominance of *Lactobacillus* genus. Most women display prevalence of one species among *L. crispatus*, *L. iners*, *L. jensenii* and *L. gasseri* (Ravel et al., 2011; Mehta et al., 2020). These taxa provide protection to the host through various mechanisms, such as lowering of vaginal pH, producing hydrogen peroxide (H_2_O_2_), synthesis of antimicrobial peptides, competition for nutrients and adhesion sites and modulation of host immune response (Parolin et al., 2015; Calonghi et al., 2017; Younes et al., 2018). However, the composition of the vaginal microbiome can vary depending upon ethnicity and exposure to different environmental factors, such as antimicrobial and non-antimicrobial drugs, diet, microbial load and exposure to different microbes in the living ecosystem (Barrientos-Duran et al., 2020).

Currently, there are several identified risk factors for PTB in which vaginal microbiome contribute substantially to the etiology. Exogenous microorganisms ephemerally colonizing the vagina have been hypothesized as an important contributor of PTB delivery. Many microorganisms isolated from the amniotic fluid or from the amniotic membrane of women who had PTB are also identified in the lower genital tract of the pregnant women (Hillier et al., 1988; Romero et al., 1989; Krohn et al., 1995; Gardella et al., 2004). The composition, diversity and dynamics of the high vaginal microbiome modulate the stability of its ecology and restrain the membership of exogenous microbial species in the vaginal niche. Dominance of non-indigenous microbial species and shift in the vaginal microbiome composition from the dominant *Lactobacillus* to a polymicrobial flora, a dysbiotic state of vaginal microbiome, substantially contribute in the pathophysiology of PTB delivery (Romero et al., 2014; Hyman et al., 2014; DiGiulio et al., 2015; Callahan et al., 2017; Klebanoff and Brotman, 2018). A number of potential microbial species, singly or in combinations, increase the risk of PTB delivery (Fettweis et al., 2019). The list of possible agents continues to expand and includes members of a number of genera, including *Gardnerella*, *Atopobium*, *Prevotella*, *Peptostreptococcus*, *Mobiluncus*, *Sneathia*, *Leptotrichia*, *Mycoplasma*, *Megasphera* and several others (Fettweis et al., 2019; Feehily et al., 2020). Recently, complex microbial assemblage, such as BVAB1, BVAB2, and BVAB3, has also been included in the continuously evolving list, as potential risk factor of PTB delivery (Fredricks et al., 2005). The metabolites or antigens produced by these microbes increases the level of local and systemic inflammatory cytokines and interstitial collagenase synthesis that have been reported to induce the PTB delivery (Stafford et al., 2017; Bukowski et al., 2017). Several molecules produced by *Lactobacillus* are linked with antimicrobial and anti-inflammatory functions and have revealed direct association with PTB risk (Stafford et al., 2017; Anton et al., 2018). However, the influences of such bacterial species and their products in the adverse birth outcomes widely vary (Amabebe and Anumba, 2018).

We recently reported the vaginal microbiome of reproductive age Indian women enrolled in the inter-disciplinary Group for Advanced Research on Birth Outcomes- A DBT India Initiative (GARBH-Ini) cohort (Mehta et al., 2020). However, we currently lack an understanding of the composition, diversity and functional repertoires of vaginal microbiome of pregnant Indian women who deliver a preterm baby. In the present study, we have investigated the differences of vaginal microbiome composition between TB and PTB samples and the genomic repertoires of the dominant *Lactobacillus* species isolated from Indian women. We studied the composition, diversity and dynamics of the vaginal microbiome by targeted sequencing of the V3-V4 hyper-variable region of the 16S rRNA gene. For functional insights, different *Lactobacillus* species associated with birth outcomes were isolated and their whole genome sequences (WGS) were decoded by shotgun sequencing. Findings of the present study enriched our knowledge to understand association of specific microbial species with birth outcomes. The WGS analysis further adds function to such microbes potentially linked with TB and PTB delivery.

## Materials and Methods

### Subject Recruitment

Translational Health Science and Technology Institute human ethics committee have approved this study (Ref.# THS 1.8.1/(30) dated 11^th^ Feb 2015). Pregnant women who visited the antenatal clinic at Gurugram Civil Hospital (GCH) before completion of 20-weeks period of gestation (POG) and provided written informed consent were enrolled in the GARBH-Ini pregnancy cohort. POG was confirmed based on ultrasonography. Vaginal swab samples were collected from the enrolled women in each trimester of pregnancy i.e., one swab each from 1^st^ (V1: <14 weeks), 2^nd^ (V2: 18-20 weeks) and 3^rd^ (V3: 26-28 weeks) trimester using sterile Catch-All™ sample collection swabs. This study was designed as a case-control study nested into the ongoing GARBH-Ini cohort (Bhatnagar et al., 2019). The cases and controls were derived from a universe of pregnant women without medical complications during pregnancy and who had singleton babies without congenital abnormalities by spontaneous delivery. The cases were women who delivered preterm. Each case was matched with a control (women who delivered at term: at 37 or more completed weeks of gestation) based on month of delivery and parity. Women with history of antibiotic usage in the 7 days prior to sampling and those who used vaginal medications were excluded. The obstetricians measured vaginal pH of the study participants using commercially available pH monitoring strip. A total of 18 preterm (delivered less than 37 completed weeks of gestation) and 20 term (gave birth at 37 or more completed weeks of gestational age) women were selected for the present study.

### High Vaginal Swab Collection

The study participants were guided to the procedure room in the GCH and positioned in the lithotomy position. The high vaginal swab samples were collected (n=115) aseptically from the midpoint of the vagina using a Cusco’s speculum and four sterile Catch-All™ sample collection swabs. The swabs were gently rubbed for ~20 sec against the mid vaginal wall. One swab placed in a sterile microcentrifuge tube that was pre-filled with 0.5 mL of sterile 50 mM Tris-1 mM EDTA buffer (pH 8.0) supplemented with nuclease inhibitors and protein-denaturing agents (Guanidinium thiocyanate) was used for microbiome study. The tubes containing swabs were vortexed rigorously for detaching microbial cells from its wall. The collected samples were then transported to the Molecular Genetics Laboratory (MGL) at Translational Health Science and Technology Institute (THSTI) within 12 hours of collection in freezing conditions (−192°C). One swab was used for microbial culturing including *Candida* species following standard microbial culture practice reported elsewhere (Pareek et al., 2019).

### Extraction of Genomic DNA From HVS Samples

Microbial genomic DNA was extracted from HVS samples using THSTI DNA extraction methods (Bag et al., 2016). Briefly, the collection buffer (Tris-EDTA) containing HVS samples were subjected to chemical, physical, and mechanical lysis procedures for disrupting microbial cells and releasing genomic DNA in the lysis buffer. Mechanical lysis was done by bead beating the samples using 0.1-mm Zirconia beads (Biospec USA) and SpeedMillPLUS bead beater (Analytic Jena, Germany). We used a circulating water bath (LAUDA cooling thermostats Alpha RA, Germany) for heat lysis of the bead beated samples at 75°C for 15 min. A denaturing organic solvent mixture phenol:chloroform and polyvinylpolypyrrolidon (Sigma-Aldrich, USA) was used to remove cellular and extracellular impurities like proteins, lipopolysaccharides and phenolic compounds. Samples were treated with RNase (New England Biolabs, USA) to remove ribonucleic acids (RNAs) from the nucleic acid pools. Finally, genomic DNA was precipitated using 90% ethanol. The precipitated DNA was washed two times with 70% ethanol to remove salt and other contaminants. Heat dried community microbial was resuspended in 100 μl sterile water. Quality and quantity of the DNA isolation from each of the samples were monitored by resolving the sample in 0.8% agarose gel. The 260/230 and 260/280 ratios were used as a secondary measure of genomic DNA purity.

### Paired-End Massively Parallel Sequencing of 16S rRNA Gene

The microbial DNA of high vaginal swab samples from 18 PTB and 20 TB mothers at all the three trimesters (V1, V2 and V3) were transported to National Institute of Biomedical Genomics (NIBMG) for amplicon based sequencing of the V3-V4 hyper-variable region of 16S rRNA gene. From the isolated DNA, the V3-V4 hyper-variable region of 16S rRNA gene was amplified using universal barcoded primer pairs: 175F (5´-CCTACGGGNGGCWGCAG-3´) and 512R (5´-GACTACHVGGGTATCTAATCC-3´) (Klindworth et al., 2013). For each sample, 4µl (>15ng/µl) microbial DNA was mixed with PCR buffer, MgSO_4_, Platinum Taq DNA Polymerase, PCR grade water, dNTPs and subjected to PCR amplification conditions of 95°C for 5 minutes, then 35 cycles of: (a) 94°C for 30 seconds, (b) 55°C for 30 seconds, (c) 68°C for 1 minute. This was finally followed by 68°C for 1 minute and lastly kept at 10°C until further processing. The negative controls collected during sample collection were processed by the same method as above. Amplified products were purified using Agencourt AMPure- XP (Beckman Coulter) paramagnetic beads and viewed by 1% agarose gel electrophoresis. Sample indexing was done by Nextera XT Index Kit (Illumina) and quantification of DNA library was performed by Qubit Flurometer using Qubit™ dsDNA HS Assay Kit (Invitrogen) and amplicon length was checked using 2100 Bioanalyzer instrument. The final libraries were pooled and sequenced using HiSeq2500 platform following 2x250 paired-end chemistry. The raw data generated was further analyzed for taxonomic classification.

### Microbiome Sequence Data Analysis

Demultiplexed FASTQ files for Read 1 (R1.fastq) and Read 2 (R2.fastq) of each sample were subjected to initial quality control based on the FastQC reports generated (http://www.bioinformatics.babraham.ac.uk/projects/fastqc) for each of the paired-end FASTQ files. The key points that were checked from FASTQC reports were whether: (a) the total number of reads in R1.fastq and R2.fastq are similar, (b) the average per base quality value >20 for all the bases in the R1 and R2 reads. The paired-end reads were then merged and sequencing primers were trimmed. The reads were filtered based on certain criteria viz., (a) average read length – 200 to 1000 bp, (b) average quality score ≥ 25, (c) maximum number of ambiguous bases ≤ 6, (d) maximum number of homopolymers≤ 6. Out of 5 negative control samples collected during sample collection, only 2 could be carried forward for further analysis since the three samples had less than 10 reads per sample. The quality filtered reads for all the samples were further analyzed by using VSEARCH v2.14.0(Rognes et al., 2016) to generate Operational Taxonomic Units (OTUs) by merging of paired reads and clustering the sequence reads at 97% identity threshold. The singletons (OTUs consisting of only one read) were removed before analysis. Removal of chimeric reads (sequences formed from two or more biological sequences joined together) both by *de novo* and reference-based methods were performed by VSEARCH on the representative sequences obtained from the OTUs. Non-chimeric representative sequences for each of the OTUs were identified and the FASTA file with such sequences were subjected to taxonomic classifications from phyla to genera levels by aligning them to Greengenes (v13_8) database (DeSantis et al., 2006) using QIIME (v1.9.1) (Caporaso et al., 2010) and an OTU table (file consisting of reads for each sample along with taxonomic assignment for each OTU) was formed. For adjusting the negative control samples, the OTUs shared by the negative controls with an average relative abundance of ≥ 1% was removed (Davis et al., 2018). The actual number of reads for each sample was then subsampled (with a bootstrap support of 100) to the minimum number of reads observed among all the samples (Mukherjee et al., 2016; Willis, 2019). Rarefaction plots were generated to confirm if (a) the number of OTUs and (b) the estimated alpha diversity (Shannon, Chao) indices were independent of the inter-individual variation of total number of reads generated for each individual i.e., reached a plateau even with minimum number of reads. Estimations of alpha (Shannon and Chao) (Chao and Shen, 2003) and beta (Jaccard and Bray-Curtis) (Bray and Curtis, 1957) diversities were performed using QIIME (Kuczynski et al., 2012) scripts. For inter-individual comparison, the number of reads that mapped to a particular taxon was normalized by the total number of reads generated for that individual to obtain the relative abundance values for each level of taxonomic hierarchy using QIIME pipeline (Caporaso et al., 2010). Species level classification was performed only on those genera that have relative abundance ≥1% in either Term or Preterm samples in any one of the three trimesters (V1/V2/V3). Those taxa that remained unclassified at the genera level were not included for species level classification. To obtain species level classification, the representative OTUs of the selected genera were aligned to the NCBI’s 16S Microbial database by BLCA tool (Gao et al., 2017) which is based on a Bayesian Lowest Common Ancestor (LCA) method. For validation of the species level taxonomy, the representative sequences were also aligned to the NCBI’s 16S Microbial database (Johnson et al., 2019) by using BLASTn (Camacho et al., 2009). The species identification was done based on ≥ 80% confidence score in BLCA and ≥ 97%% sequence identity in BLAST.

### Statistical Analysis of Vaginal Microbial Taxa Abundances and Diversity Indices Between Term and Preterm Mothers

Differences in POG at delivery and maternal age at conception between term and preterm delivering women were compared by unpaired two-tailed t-test. The microbial taxa identified by analyzing 16S rRNA gene sequencing data were compared between term and preterm samples in each of the three trimesters V1, V2 and V3. The identification of the core microbiome from the total number of taxa was based on fulfilment of the following conditions by any of the two groups of mothers (i.e., delivering term or preterm) and at any of the three trimesters (V1/V2/V3): (a) average relative abundance ≥0.1%, and (b) presence in at least 50% of individuals in the group. Non-parametric Wilcoxon rank-sum test (two-tailed) was performed using the R command (wilcox.test, paired = FALSE) to identify those taxa that are significantly associated with PTB in each of the three trimesters. The alpha and beta diversity indices were also compared between term and preterm samples across all the three trimesters and p-value ≤0.05 was considered significant.

We have carried out Linear Mixed Effects model-based analysis to investigate the fixed effects of Birth Type (preterm/term) and Gestation Time (first/second/third trimesters) on the relative abundance of the predominant genera. We have considered those genera as predominant that have mean relative abundance ≥1% in either TB or PTB group in any of the three trimesters. Species level data were available only for those genera and were also included in this analysis. The current analysis was performed using q2-longitudinal (Bokulich et al., 2018).

### Isolation and Identification of *Lactobacillus* Species

For the isolation of *Lactobacillus* species the swab samples were collected from ten term vaginal samples. The swabs were resuspended in the Amies transport medium and transported to the MGL at THSTI in anaerobic condition within 6 hours of sample collection. We used 50 μl of transport medium to isolate discrete colonies onto a de Man-Rogosa-Sharpe (MRS) agar plate (Sigma-Aldrich, Carlsbad, CA). The plates were incubated in an anaerobic workstation (Whitley A95TG, UK) at 37°C for 48 hrs. Distinct colonies were picked up and grown in MRS broth under anaerobic growth conditions at 37°C for 48 hrs. Bacterial isolates grown in the MRS medium were subjected to genomic DNA extraction and amplification of complete 16S rRNA gene followed by DNA sequencing. Bacterial isolates with more than 97% sequence identity of their 16S rRNA gene with the reported *Lactobacillus* genus were selected for further study.

### Whole Genome Sequencing Assembly and Annotation

Whole genome sequencing of the confirmed *Lactobacillus* genus was done adopting shotgun sequencing using a high-throughput Illumina MiSeq sequencing platform (Illumina, Inc., USA) at THSTI. Approximately, 100 ng of pure genomic DNA was used for DNA fragmentation and library preparation and pair-end sequencing using Nextera XT DNA Library preparation kit (Illumina, Inc., USA). FastQC and Trimmomatic programs were used to review the quality of raw reads and remove adapter sequences and low quality reads. An average of 4,03,396 clean quality filtered reads were used to generate the draft genome of 10 *Lactobacillus* isolates belonging to two different species. The average sequencing coverage of the genomes was ~34.63 times. The cleaned pair-end reads were used for genome assembly using Unicycler pipeline (Wick et al., 2017). After assembly, all the contigs of the genome were annotated by Rapid Annotation Subsystem Technology (RAST) automated annotation pipeline (Aziz et al., 2008). Annotated proteins were further confirmed by comparing their sequence homology with the reported proteins publicly available in the Protein database (PDB). Around 98% genes predicted to encode proteins were also available in the PDB. Whole genome sequences of all the *Lactobacillus* strains will be available immediately after acceptance of the article.

### Estimation of Core- and Pan-Genome

A total of 42 whole genome sequences of three *Lactobacillus* species viz. *L. crispatus*, *L. iners* and *L. gasseri* were considered for analysis of highly conserved stable core genome and total genomic contents. All orthologous gene clusters were identified by get_homologues (Contreras-Moreira and Vinuesa, 2013) pipeline by applying following parameters for identification and clustering CDS into orthologous groups: (i) -E < 1e-05 for protein searches by protein Basic Local Alignment Search Tool (BLAST) (Camacho et al., 2009) and 40% of sequence identity with 75% coverage in BLAST pairwise alignments. Ortho Markov Cluster algorithm (OMCL) (Enright et al., 2002; Li et al., 2003) with –t 0 was used to find core-genome and the pan-genome.

### Phylogenetic Analysis

Comparative genomics were performed by pan- and core- genome based phylogenetic analysis. Based on all the orthologues gene clusters, a present/absent matrix was created to draw a pan-genome based phylogeny. For core genome based phylogeny, only those gene clusters were considered that are present among all the genomes. Some core gene clusters contain inparalogs, and then the longest sequence was chosen for further analysis. Consequently, each core gene cluster contains only single gene from each genome. All gene clusters were aligned by Clustal Omega program (Sievers and Higgins, 2014). Those genes belonging to the same genomes that were concatenated to make a single long sequence. In this way, same numbers of concatenated sequence were created as numbers of genomes were taken in this study. Finally, the aligned sequences were used as input to IQ-TREE (Lam-Tung et al., 2015) program to generate phylogenetic tree based on maximum likelihood method with automatic chosen the best-fit by IQ-TREE server. The branch tree support analysis was performed by 1000 bootstrap and SH-aLRT branch test. Finally, the generated tree was annotated by iTOL server with other metadata (Letunic and Bork, 2019).

### DNA Binding Domain and Secretory Signal Motif Analysis

The hypothetical proteins present in the genome of the different lactobacilli were used for finding the DNA binding domain using the Conserved Domain Database (CDD) keeping 0.0001 as the threshold parameter (Lu et al., 2020). 50% bitscore was kept as the criteria for selecting the conserved DNA binding domain. The signal peptide was searched using SecretomeP 2.0 server (Bendtsen et al., 2005). This server produces ab initio predictions of non-classical peptide sequences. For bacterial sequences the SecP value was ≥ 0.5. Based on this criterion the signal peptide sequences were sorted out. Next we used PSORT server (Horton et al., 2007), which is a computer program for the prediction of protein localization sites in cells (Horton et al., 2007). It converts protein amino acid sequences into numerical localization features; based on sorting signals, amino acid composition and functional motifs such as DNA-binding motifs. Finally, it reports the possibility for the input protein to be localized at each candidate site with additional information.

### Availability of Nucleotide Sequences

Whole genome sequences of all the 10 *Lactobacillus* strains are deposited in the National Center for Biotechnology Information (NCBI) GenBank (Submission ID is SUB9505031). Accession numbers for all the genome sequences will be communicated shortly. Metadata and 16S rRNA gene sequences are submitted to the European Nucleotide Archive (Study accession number is: PRJEB43005).

## Results

### Characteristics of Study Participants

The median age of the participants (n=38) included in this study was 22 years (interquartile range (IQR): 21, 25). Significant differences in POG at delivery [TB- (mean ± s.d.) 38.8 ± 1.2 weeks; PTB- (mean ± s.d.) 35 ± 2.4 weeks; p- value= 6.68x10^-6^] were observed between term and preterm samples. However, the maternal age at conception (TB- avg. 22.72 ± 3.2 yrs; PTB- avg. 23.5 ± 4.1 yrs; p- value = 0.5) between term and preterm was not significant. Nearly one-third of the women were underweight and about 15% were overweight or obese. Two participants had complaints of vaginal discharge and none had bleeding per vaginum. The median vaginal pH was 5 and the Nugent’s score was 4 (IQR: 3,5). Five of the participants had *Candida* species grown in the culture of their vaginal fluid. Further detailed characteristics are provided in **Table 1**.

**Table 1:** Relevant characteristics of the enrolled study participants (n=38) of the Interdisciplinary Group for Advanced Research on Birth Outcomes-DBT India Initiative (GARBH-Ini) Cohort, Haryana, India.

Characteristics	Term(n=20) n (%) or Median (IQR)	Preterm(n=18) n (%) or Median (IQR)
***Clinical and demographic***		
Maternal age (year)	23 (21,26)	22 (20,25)
Weight at enrollment (kg)	46.1 (40.9,53.5)	46.0 (41.5,50.2)
BMI at enrollment	19.61 (18.52,22.49)	19.10 (17.48,20.62)
BMI category		
Underweight	5 (25.00%)	8 (44.44%)
Normal	11 (55.00%)	8 (44.44%)
Overweight	2 (10.00%)	0 (0.00%)
Obese	2 (10.00%)	2 (11.11%)
Gravidity		
Primigravida	6 (30.00%)	7 (38.89%)
Multigravida	14 (70.00%)	11 (61.11%)
History of vaginal discharge		
Present	1 (5.00%)	1 (5.56%)
Absent	19 (95.00%)	17 (94.44%)
History of bleeding per vagina		
Present	0 (0.00%)	0 (0.00%)
Absent	20 (100.00%)	18 (100.00%)
History of diarrhea		
Present	0 (0.00%)	1 (5.56%)
Absent	20 (100.00%)	17 (94.44%)
Socioeconomic status		
Upper middle class	2 (10.00%)	3 (16.67%)
Lower middle class	2 (10.00%)	6 (33.33%)
Upper lower class	15 (75.00%)	9 (50.00%)
Lower class	1 (5.00%)	0 (0.00%)
Toilet usage		
Flush/pour flush toilet	19 (95.00%)	17 (94.44%)
Bucket latrine	1 (5.00%)	1 (5.56%)
***Laboratory characteristics***		
POG at high vaginal		
At enrolment	11w3d (9w2d,12w5d))	10w4d (7w0d,12w5d)
At Visit 2	19w4d (18w1d,19w5d)	19w5d (18w2d,19w6d)
At Visit 3	26w3d (26w2d,26w4d)	26w2d (26w1d,26w4d)
Birth outcomes		
Normal vaginal	18 (90.00%)	17 (94.44%)
Caesarean	2 (10.00%)	1 (5.56%)
Nugent score		
At enrolment	4 (3,5)	4 (3,5)
At Visit 2	4 (3,5)	4 (3,5)
At Visit 3	4 (2,5)	3 (2,4)
Vaginal pH at sampling	5 (5,5)	5 (5,5)
Antibiotic intake at sampling	0 (0.00%)	0 (0.00%)
Microbial culture result for HVS samples (N=37)		
**At enrolment**		
Candida species	2 (10.53%)	3 (16.67%)
Non-pathogenic microbes	16 (84.21%)	14 (77.77%)
Sterile	1 (5.26%)	1 (5.56%)
** At Visit 2**		
Candida species	5 (26.31%)	2 (11.11%)
Non-pathogenic microbes	11 (57.89%)	13 (72.22%)
Sterile	2 (10.53%)	3 (16.67%)
Escherichia coli	1 (5.26%)	0 (0.00%)
** At Visit 3**		
Candida species	3 (15.00%)	0 (0.00%)
Non-pathogenic microbes	13 (65.00%)	12 (70.59%)
Sterile	4 (20.00%)	5 (29.41%)

### Diversity Indices of the Core Microbial Taxa in the Vaginal Milieu of Women With Term and Preterm Delivery

A total of 115 high vaginal swab samples collected from three different time points during pregnancy were selected for the 16S rRNA gene sequencing based analysis. The average number of paired-end reads were reduced to 0.86 million from 0.95 million after initial QA/QC and chimera removal (**Supplementary Table S1**).

**Figure 1:**
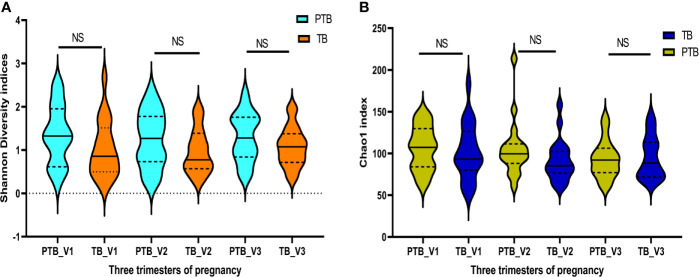
Diversity indices: Intra- individual diversity (alpha diversity) is not significantly different between preterm and term delivering mothers. **(A)** Shannon diversity indices, **(B)** Chao1 indices, at different trimester of pregnancy. ns, non-significant.

Quality filtered non-chimeric reads were clustered into bins based on 97% sequence identity which resulted in 949 OTUs after removal of singletons and OTUs (with relative abundance ≥1%) shared with negative control samples that passed the initial QA/QC step. The sequences were rarefied by subsampling to the minimum number of reads per sample i.e. 262637 sequences per sample (Rarefaction curve in **Supplementary Figure 1**). Alpha diversity indices such as Shannon (PTB - 1.35 ± 0.65, TB - 1.02 ± 0.63; p > 0.5) and Chao1 (PTB - 105.5 ± 93.1, TB - 91.38 ± 24.2; p > 0.05) were found to be higher in preterm samples compared to term samples in all the three trimesters of pregnancy but not statistically significant (**Figures 1A, B**).

A total of 16 bacterial phyla and 217 bacterial genera were identified in the term whereas 17 bacterial phyla and 244 bacterial genera were identified in the preterm samples. The core taxa consisted of five phyla namely, *Actinobacteria* (PTB - 6.6%, TB - 4.2%), *Bacteroidetes* (PTB - 0.66%, TB - 0.35%), *Firmicutes* (PTB - 39.02%, TB - 42.5%), *Proteobacteria* (PTB - 53.1%, TB - 52.9%) and *Fusobacteria* (PTB - 0.63%, TB - 0.0009%). Twenty genera were identified as the core genera, which include *Lactobacillus* (PTB - 37.6%, TB - 41.6%), *Enterobacter* (PTB - 36.7%, TB - 40.1%), unclassified genus of *Pseudomonadaceae* family (PTB - 11.6%, TB - 10.4%), *Gardnerella* (PTB - 5.34%, TB - 2.4%) and *Halomonas* (PTB - 2.37%, TB -1.05%) as the top five abundant genera in all the samples. The core vaginal microbiome between term and preterm samples were analyzed and compared. The relative abundances of the core genera in term and preterm samples in all the three trimesters are given in **Table 2** and with species level data in **Supplementary Table S2**.

**Table 2:** Core Vaginal Microbiome in Term and Preterm samples in all the three trimesters.

Core Taxa		Mean relative abundance of core genera in Term and Preterm samples in all the three trimesters								
		1^st^ trimester (V1)			2^nd^ trimester (V2)			3^rd^ trimester (V3)		
**Core Phyla**	**Core Genera**	**V1_PTB**	**V1_TB**	**P value**	**V2_PTB**	**V2_TB**	**P value**	**V3_PTB**	**V3_TB**	**P value**
**Actinobacteria**	*Corynebacterium*	0.17	0.06	0.92	0.01	0.01	0.67	0.006	0.01	0.74
	f_Bifidobacteriaceae ;g_unclassified	7.18	3.09	0.23	4.20	0.87	**0.01**	4.65	3.26	0.43
	f_Coriobacteriaceae ;g_unclassified	1.80	2.77	0.68	0.96	1.78	0.07	0.58	0.68	0.53
**Bacteroidetes**	*Prevotella*	0.22	0.02	0.12	0.06	0.007	0.08	0.45	0.09	0.41
	*Elizabethkingia*	0.01	0.08	0.44	0.12	0.05	0.51	0.24	0.24	0.55
	*Sphingobacterium*	0.33	0.35	0.53	0.09	0.07	0.59	0.42	0.09	0.85
**Firmicutes**	*Aerococcus*	0.09	0.0002	0.09	0.82	3.6x10^-05^	**0.003**	0.03	0.03	0.33
	*Lactobacillus*	39.71	40.20	0.74	38.22	43.21	0.73	34.95	41.28	0.50
	*Streptococcus*	0.005	2.21	0.22	0.004	0.005	0.93	0.002	0.004	0.78
	*Clostridium*	0.07	0.05	0.23	0.10	0.003	0.12	0.07	0.02	0.33
	*Megasphaera*	1.45	0.001	**0.02**	0.34	0.004	0.12	0.51	0.001	0.11
	*Veillonella*	0.03	0.004	0.35	0.21	0.003	0.06	0.007	0.03	0.54
**Fusobacteria**	*Sneathia*	0.01	0.0008	0.82	1.54	0.0004	**0.00001**	0.34	0.0008	**0.04**
**Proteobacteria**	*Ochrobactrum*	0.05	0.002	0.35	0.001	0.8	0.56	0.009	0.0007	0.65
	*Achromobacter*	0.10	0.20	1.00	0.20	0.28	0.68	2.32	0.034	0.91
	*Ralstonia*	0.75	0.61	0.76	1.74	0.58	0.94	1.12	0.59	0.54
	f_Enterobacteriaceae;g_other	35.74	41.79	0.57	43.79	38.73	0.43	30.60	39.73	0.43
	f_Enterobacteriaceae;g_unclassified	0.17	0.20	0.48	0.175	0.178	0.60	0.10	0.16	0.48
	*Halomonas*	1.80	1.49	0.36	2.91	1.19	0.69	2.40	0.48	0.35
	f_Pseudomonadaceae;g_unclassified	9.92	6.51	0.97	4.07	11.84	0.89	20.72	12.86	0.35

p-values in bold letters are considered to be statistically significant (p<0.05).

### Differential Abundance of *L. crispatus, L. gasseri and L. iners* in Term and Preterm Mothers

**Figure 2:**
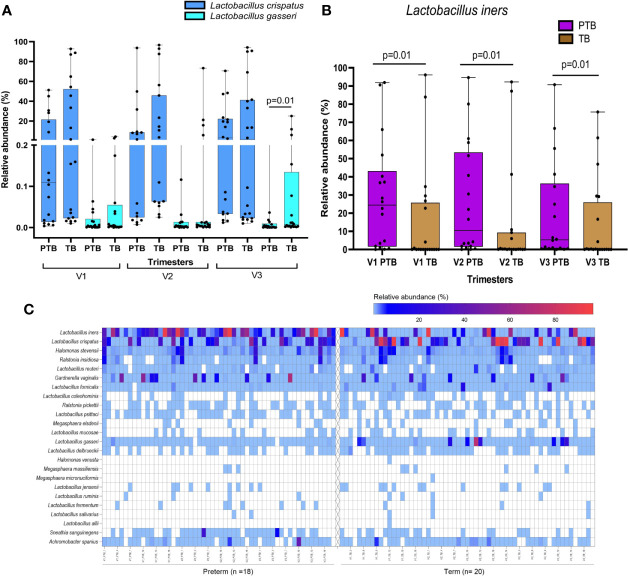
Box plots showing differential abundance of *L. crispatus, L. gasseri* **(A)**, and *L. iners* **(B)** between TB and PTB samples. *L. gasseri* was found to be significantly higher in the TB compared to PTB samples (3rd trimester only). *L. iners* was found to be significantly higher in PTB compared to TB samples in all the trimesters. **(C)** Heatmap representing species level composition of those genera with mean relative abundance >1% in either term or preterm group in any of the three trimesters. Left panel of the heatmap is for the PTB samples while the right side is for the TB samples.

*Lactobacillus* is the most abundant genus in the vaginal milieu of reproductive age women globally. We have compared relative abundance of a phylum/genus/species between mothers who delivered term or preterm and between trimesters within a group of mothers. These comparisons resulted in a large number of tests. However, we have not performed corrections for multiple testing primarily because these tests are related and cannot be viewed as independent test, which is assumed in the methods used for multiple testing corrections. In view of this, we suggest that our results when declared as significant be considered as tentative. These are discoveries that require validation in future studies to be conducted by us or by other investigators. In the present study, we observed that the abundance of genus *Lactobacillus* is similar in both term (41.6%) and preterm (37.6%) delivering mothers. However, several species of *Lactobacillus* reside in the reproductive tract. A total of 19 species of *Lactobacillus* were identified among term and preterm delivering women with *L. crispatus*, *L. iners, L. gasseri*, *L. fornicalis* and *L. delbrueckii* as the top five abundant species. The species level abundance of different lactobacilli between term and preterm mothers reveal distinct patterns. Women delivering at term (23.5%) were found to harbor higher abundance of *L. crispatus* compared to women delivering preterm (9.8), although not statistically significant (p>0.05). We have found that in the third trimester abundance of *L. gasseri* is significantly higher in the HVS samples of women who delivered term baby compared to those who delivered preterm (TB: 2.163 ± 6.212, PTB: 0.023 ± 0.074; p=0.01, **Figure 2**). *L. iners* was found to be significantly higher in preterm samples in all the three trimesters compared to the term samples (p ≤ 0.02; **Figure 2**), which gives us an insight to predict the risk of preterm delivery in pregnant women and the type of *Lactobacillus* species predominant in their vaginal microbiome.

### Analysis of Microbial Community State Types in the Vaginal Microbiome of Term and Preterm Samples

The vaginal microbiome profiles in term and preterm samples were further assigned to Community State Types (CST) based on the dominant species abundance as reported previously (De Seta et al., 2019; Fettweis et al., 2019). Our analysis revealed four major CSTs in the vaginal microbiota of the Indian women. CST-I, CST-II, and CST-III are dominated by *L. crispatus*, *L. gasseri* and *L. iners*, respectively. CST IV is dominated by non-*Lactobacillus* species. CST-I was predominant in the TB samples (one-tailed p-values were significant only in 2^nd^ trimester for CST-I: 0.02; equality of proportions test between PTB and TB were performed for each trimester) whereas CST-III and CST-IV were predominant in the PTB samples (one-tailed p-values were significant only in 2^nd^ trimester for CST-III: 0.03 and CST-IV: 0.03; equality of proportions test between PTB and TB were performed for each trimester). In addition, we observed that CST-II was present in term samples only but completely absent in the preterm samples (**Table 3**).

**Table 3:** Result for CST distribution in term and preterm samples.

Dominant taxa	CST Type	V1_PTB	V1_TB	V2_PTB	V2_TB	V3_PTB	V3_TB
*L. crispatus*	CST I	6(30%)	9(45%)	3(16.6%)	9(47.3%)	7(38.8%)	10(50%)
*L. gasseri*	CST II	0(0%)	1(5%)	0(0%)	4(21%)	0(0%)	2(10%)
*L. iners*	CST III	9(50%)	5(25%)	10(55.5%)	5(26.3%)	8(44.4%)	5(25%)
*Non- Lactobacillus*	CST IV	3(16.6%)	5(25%)	5(27.7%)	1(5.2%)	3(16.6%)	3(15%)

Total four CSTs were identified in our data set namely CST I, CST II, CST III and CST IV which is dominated *by L. crispatus, L.gasseri, L. iners, and* Non*-Lactobacillus* respectively.

### Non-*Lactobacillus* Bacterial Taxa Associated With Preterm Birth

Predominance of a mixture of facultative anaerobic bacteria such as *Gardnerella, Sneathia* and *Megasphaera* are inversely correlated with the abundance of *L. crispatus* and some other lactobacilli. Among the 20 core genera (mean relative abundance ≥0.1% and present in 50% samples in any group), species level classification was done for 7 genera (mean relative abundances ≥ 1% in any one group and successfully assigned at the genera level) (**Supplementary Table S2**). We observed that the *Sneathia sanguinegens* (PTB: 1.54%, TB: 0.34%) abundance is significantly (p-value < 0.05) higher in 2^nd^ and 3^rd^ trimesters of preterm delivering women. The prevalence of *Gardnerella vaginalis* (PTB: 4.44%, TB: 0.87%) is also significantly (p- value <0.05) higher in preterm delivering women at 2^nd^ trimester in our study. Abundance of *Megasphaera* sp. (PTB: 1.45%, TB: 0.0007%) is significantly (p- value < 0.05) higher in 1^st^ trimester of preterm delivering mothers (**Table 4**). No particular species of *Megasphaera* sp. was found to be significantly different between TB and PTB samples.

**Table 4:** Non-Lactobacillus sp. in vaginal environment.

Significant Taxa	Mean Relative abundance of Species in Term and Preterm samples in all the three trimesters								
	V1_PTB (%)	V1_TB (%)	P value	V2_PTB (%)	V2_TB (%)	P value	V3_PTB (%)	V3_TB (%)	P value
*Gardnerella vaginalis*	7.19	3.09	0.23	4.44	0.87	**0.01**	4.64	3.26	0.43
*Megasphaera* sp.*	1.45	0.001	**0.02**	0.34	0.004	0.12	0.51	0.001	0.1
*Sneathia sanguinegens*	0.01	0.0008	0.82	1.54	0.0004	**0.00001**	0.34	0.0008	**0.04**

*Sneathia sanguinegens* is significantly (p-value < 0.05) higher in 2^nd^ and 3^rd^ trimesters, *Gardnerella vaginalis* is significantly (p- value <0.05) higher at 2^nd^ trimester and *Megasphaera* sp. is significantly (p-value <0.05) higher in 1^st^ trimester of preterm samples compared to term samples. *No particular species of genus Megasphaera was found to be significantly different between TB and PTB samples.

p-values in bold letters are considered to be statistically significant (p<0.05).

### Longitudinal Analysis of Selected Taxa Using q2-Longitudinal

As mentioned in the previous section, species level data were generated for 7 predominant genera among 20 core genera identified in the present study. Statistical analysis of these 7 predominant genera and their species was carried out to identify significance of variation over the time course of pregnancy using q2-longitudinal. For most microbial groups, none of the effects turned out to be significant. The effects of birth type and gestation time were significant for the genera *Lactobacillus* and *Megasphaera*. Within *Lactobacillus*, these effects were also significant for the species *L. iners* and *L. psittaci*. For the two genera (*Lactobacillus* and *Megasphaera*), the trends of change in abundance over gestational time were significantly dissimilar between mothers who gave birth at term and at preterm (interaction effects between birth type and gestational time were significant for these two genera). Detailed results are provided in (**Supplementary Table S3**).

### Isolation, Identification and Characterization of Dominant *Lactobacillus* Species Associated With Birth Outcomes

Lactobacilli have long been known as beneficial members of the vaginal microbiota and play an important protective role against microbial infections and reduce the risk of PTB. Different members of lactobacilli are highly diverse and phylogenetically heterogeneous with about more than 170 species being the native members of the vaginal and gastrointestinal tract microbiomes(Goldstein et al., 2015). We observed that the two dominant *Lactobacillus* species (*L. crispatus*, and *L. gasseri*) residing in the vaginal milieu of Indian women are associated with term while *L. iners* in the vaginal milieu of Indian women are associated with PTB outcomes. For functional insights, we have isolated three *Lactobacillus* species from the HVS of the enrolled women in the GARBH-Ini cohort (Bhatnagar et al., 2019). For isolation, we used *Lactobacillus* specific growth medium (MRS) and anaerobic growth conditions as described in the method section. Isolated lactobacilli were further confirmed by complete 16S rRNA gene sequencing using Sangar dideoxy chain termination sequencing technology. We set 97.5% sequence identity and 100% coverage to assign specific taxa for each of the isolates.

### Genomic Repertoires of Abundant *Lactobacillus* Species

Since, the relative abundance of *L. crispatus, L. gasseri* and *L. iners* are high compared to the other bacterial species of the vaginal microbiota and these three species are known to play an important role in birth outcomes, we focused to isolate multiple isolates belonging to these three *Lactobacillus* species. We isolated 14 colony-forming units (CFU) and decoded their whole genome sequences by adopting shotgun sequencing to explore genomic repertoires and adding functional insights. Genome sequences of 4 of the 14 isolates were reported in our previous study (Mehta et al., 2020). Genome sequences of all the 14 isolates i.e. *L. crispatus* (n = 7), *L. gasseri* (n = 6) and *L*. *iners* (n = 1) were deposited in the National Center for Biotechnology Information (NCBI). The genome sequences were subjected to automated annotation using NCBI prokaryotic genome annotation pipeline (Tatusova et al., 2016) or Rapid Annotation using Subsystem Technology (RAST) server (Aziz et al., 2008). Major focus was given to identify and analyze functions that potentially contribute in bacteriocin and lysin productions, which are secretory in nature. Relevant features of the *Lactobacillus* genomes isolated from the GARBH-Ini cohort are mentioned in **Table 5**. The numbers of ORFs in the fourteen genomes differ from 1178 to 2249. All the fourteen genomes harbored several enzyme-encoding genes linked with DNA mobility and site-specific recombination proteins like tyrosine recombinases, transposases and integrases. These functions are often physically linked with mobile genetic elements (MGEs) and play important role in the acquisition and dissemination of fitness traits, metabolic enzymes, antimicrobial resistance, antimicrobial peptides and other functions that modulates microbial composition and inflammation in the vaginal milieu. Genetic components associated with CRISPR-Cas were also prevalent in the genome of *L. crispatus, L. gasseri* and *L. iners*. Functions conferring resistance to different antibiotics including β-lactamases, multidrug and toxin extrusion (MATE) family efflux pump, multidrug resistance efflux pumps, RND multidrug efflux transporter, ABC transporters and major facilitator superfamily (MFS) multidrug efflux transporter are present in the genome of all three *Lactobacillus* species. Several functions linked with phage replication, integration, and mobility is detected in the *L. crispatus, L. gasseri* and *L. iners* genomes. The genome of *L. crispatus* and *L. gasseri* harbors phage integrase (tyrosine recombinase) that mediate the integration of a bacteriophage into its chromosome. Similar phages are also present in the genome of other *Lactobacillus* (Acc. No. WP_060791041.1, WP_057726712.1). However, we didn’t observe any phage integrase in the genome of *L. iners.*

**Table 5:** General genomic features of 14 Lactobacilli assembled genomes.

S.N.	Species name	Genome size (bp)	Contig Number	N50	GC percent	CDS
1	*Lactobacillus crispatus 5.1*	2191964	34	103210	36.7	2249
2	*Lactobacillus crispatus 7.2*	2188668	36	96587	37.7	2248
3	*Lactobacillus crispatus 8.2*	2190096	35	103043	36.7	2248
4	*Lactobacillus crispatus 9.2*	2049433	155	19237	37	2213
5	*Lactobacillus crispatus 10.2*	2069648	165	19122	37	2233
6	*Lactobacillus crispatus Indica1*	1641433	59	33495	37.32	1596
7	*Lactobacillus crispatus Indica2*	2209487	39	153263	36.48	2024
8	*Lactobacillus gasseri 221*	1457943	106	23274	35.1	1462
9	*Lactobacillus gasseri 219*	1542387	64	51720	35	1543
10	*Lactobacillus gasseri 218*	1528392	61	51720	35	1530
11	*Lactobacillus gasseri 217*	1528051	61	51720	35	1531
12	*Lactobacillus gasseri 216*	1561727	50	57861	35	1583
13	*Lactobacillus gasseri Indica1*	2096244	5	1845454	34.92	1547
14	*Lactobacillus iners Indica1*	1331119	1	1331119	33.2	1178

All the three genomes of *Lactobacillus* encode ribosomally synthesized antibacterial peptide–related functions and permease component to protect the vaginal milieu from the invasion of non-indigenous microbiota. A protein that confers tolerance to colicin V is also present in the genome of *L. gasseri*. The genome of *L. crispatus* encodes bacteriocin helveticin and helveticin J, bacteriocin transporters, bacteriocin peptide. These were also observed in the genome of other *L. crispatus* strains (Acc. No. WP_005729773.1, WP_181577227.1, WP_150399102.1). It is known that bacteriocins are antimicrobial peptides and they are mostly active against closely related bacterial species. The genome of *L. crispatus* contains several other lysins, including enterolysin A, autolysin, streptolysin, thermolysin. Phage lysin is present in the genome of both *L. crispatus* and *L. iners* but not in the genome of *L. gasseri*. The bacteriocins produced by the different *Lactobacillus* species help in reducing bacterial diversity in the vaginal milieu and also decrease level of microbial origin inflammatory compounds by inhibiting growth of several Gram-negative bacteria.

Two out of seven genomes of *L. crispatus* are also equipped with several functions including conjugation protein, TraG/TraD that directly facilitate horizontal gene transfer (HGT) and bacterial evolution. The genome of *L. crispatus* harbors type-IIA clustered regularly interspaced short palindromic repeats (CRISPR) and multiple CRISPR spacers. CRISPR-Cas system is reported to be an important bacterial defense mechanism, which provides adaptive immunity to bacteria against invasion of MGEs like phages and plasmids (Bhaya et al., 2011). The type-IIA CRISPR-Cas system is the most dominant among lactobacilli. Interestingly, CRISPR-Cas system is present in *L*. *crispatus* but not found in the genome of *L. gasseri* and *L. iners*.

### Pan- and Core-Genome of *Lactobacillus*

Along with the whole genome sequences of 14 *Lactobacillus* strains isolated from the study subjects enrolled in the GARBH-Ini cohort, we also included 28 additional genome sequences of *L. crispatus* (n = 10), *L. iners* (n = 13) and *L. gasseri* (n = 5) publicly available in the NCBI genome database for comparative genome analysis.

**Figure 3:**
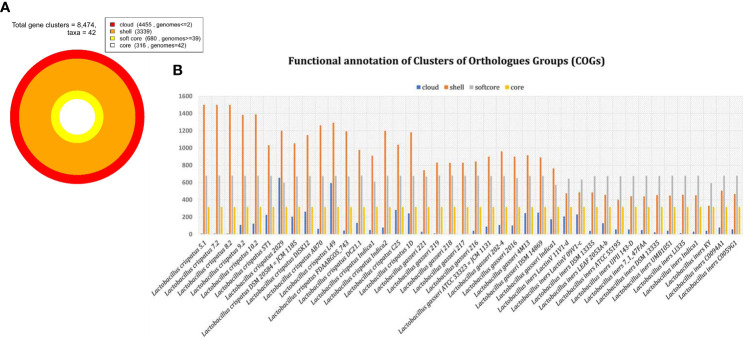
Distribution of orthologous gene clusters. **(A)** Total gene cluster and its distribution in cloud, shell, soft core and core part of genome among 42 genomes. **(B)** The bar graph of individual genome in cloud, shell, soft core and core genome.

Total 8474 orthologous gene clusters were identified from 42 genomes and considered as pan genome (**Figure 3**). Of them, 316 gene clusters were present in all 42 genomes. Therefore, 316 gene clusters were considered as core part of 42 genomes. The core gene clusters usually involved in fundamental and essential cellular processes (Carlos Guimaraes et al., 2015). We observed that 680 gene clusters are present in ≥95% of the genome included in the present analysis and is referred as soft core of 42 genomes. 3339 gene clusters were considered as shell genome as they are present more than 2 genomes but less than 95% of the genomes. The softcore and shell part of pan-genome are collectively considered as accessory or dispensable genome to perform specific functions related to adapt in different niches. It contains virulence factors, antibiotic resistance genes and different metabolic enzymes important for the survival of the microorganism in specific environments (Mira et al., 2010). 4450 gene clusters were identified as cloud genes. This subset of pan-genome is called species or strain specific genes as present in either or less than 2 genomes. These gene clusters are normally acquired by horizontal gene transfer process to get competitive advantage over those strains that do not have (Muzzi et al., 2007; Penn et al., 2009). About 52% orthologues gene clusters belong to cloud genome. It reflects more than half of pan-genome is species specific or acquired genome.

**Figure 4:**
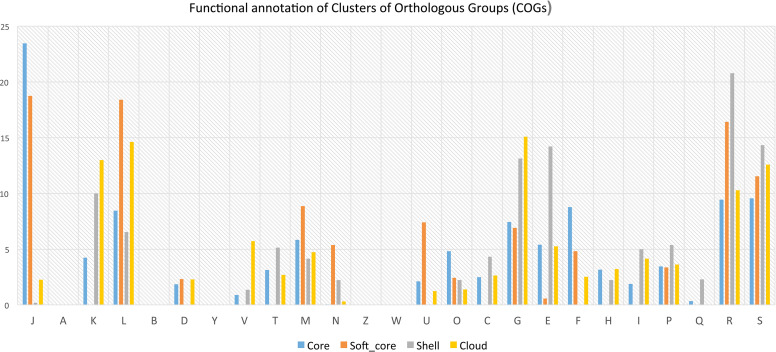
Bar plot showing distribution of COGs in core, softcore, shell and cloud subset of pan-genome J: Translation, ribosomal structure and biogenesis, A: RNA processing and modification, K: Transcription, L: Replication, recombination and repair, B: Chromatin structure and dynamics, D: Cell cycle control, cell division, chromosome partitioning, Y: Nuclear structure, V: Defense mechanisms, T: Signal transduction mechanisms, M: Cell wall/membrane/envelope biogenesis, N: Cell motility, Z: Cytoskeleton, W: Extracellular structures,U: Intracellular trafficking, secretion, and vesicular transport, O: Posttranslational modification, protein turnover, chaperones, C: Energy production and conversion, G: Carbohydrate transport and metabolism, E: Amino acid transport and metabolism, F: Nucleotide transport and metabolism, H: Coenzyme transport and metabolism, I: Lipid transport and metabolism, >P:Inorganic ion transport and metabolism, Q: Secondary metabolites biosynthesis, transport and catabolism, R: General function prediction only, S: Function unknown.

Functional annotation of pan-genome has revealed that fundamental cellular process such as DNA replication, transcription, translation, ribosomal biogenesis (class: J); nucleotide transport & metabolism, recombination and repair (class: F); and several others are most abundant in core and soft-core part of the genomes (**Figure 4**). Several functions associated with cell division, chromosome partitioning (class: D), cell wall/membrane/envelope biogenesis (class: M) are also abundant in the core genome (**Figure 4**). It has also been observed that some of above-mentioned functional classes are present in the shell and cloud genomes. The gene clusters for such additional metabolic functions are possibly acquired through HGT. Functions that provide fitness and growth advantages like carbohydrate transport and metabolism (class: G); amino acid transport and metabolism (E), coenzyme transport and metabolism (class: H); lipid transport and metabolism (class: I), inorganic ion transport and metabolism (class: P); secondary metabolite biosynthesis pathways, transport and catabolism (class: Q) their abundance (H) are also relatively high in the shell and cloud genomes. These functions are part of the shell and cloud genomes and they perform important functions and help *Lactobacillus* to compete with other microorganisms living in the same vaginal milieu.

### Pan- and Core-Base Phylogeny

**Figure 5:**
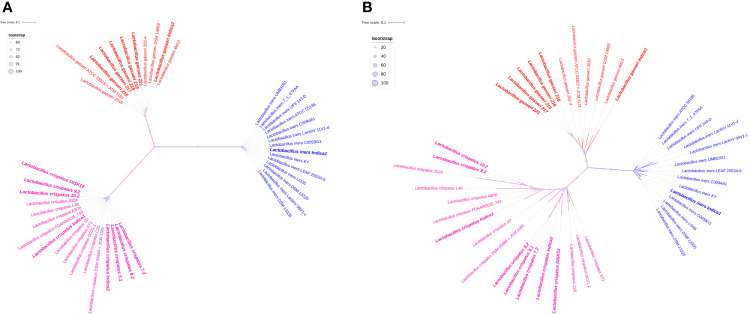
Pan- and core-genome based unrooted phylogenetic trees. **(A)** Core-genome based phylogeny. **(B)** Pan-genome based phylogeny. All three clades of phylogeny have been shown red, green and blue colors. Branch length of each leaf has not shown in number but it is proportional to divergence from last common ancestor. Bootstrap values of pan genome based phylogeny have range to ≥20 to 100. In case of core genome, boot strap value range to ≥64 to 100. It has been shown in light blue circle. The radius of the circle ranges from 5 to 15 pixels in both phylogeny trees. Indian strains of *Lactobacillus* have been shown in bold.

Pan- and core genome based phylogeny have shown similar clustering pattern (**Figure 5**). Both the trees revealed that there are three clades and each clade have made up with same species. Pan-genome based phylogeny reveals that *L. crispatus* clade is more dispersed than other two clades. The branch length of each species is different and the most species has not originated from a single common ancestor. All 7 Indian origin *L. crispatus* are distributed in two groups. It reflects that a significant number of genes may have sporadic distribution or accumulated mutations. This might lead to the significant intra-species heterogeneity in *L. crispatus* living in the same environment. Complete genome sequencing of additional *L. crispatus* isolates can help for a definite conclusion. The phylogenetic clade containing *L. gasseri* is less diverse than *L. crispatus.* All Indian *L. gasseri* strains are very close to each other except *L. gasseri* indica1. The *L. iners* clade is the most conserved among all three *Lactobacillus* species. It reflects that the pan-genome of *L. iners* is much conserved and a similar set of genes may be present in all the *L. iners* strains analyzed in the present study. The *L. iners* isolated from the GARBH-Ini cohort is very close to the *L. iners* C0059G1 isolated from Baltimore USA in 2019 (**Supplementary Table S4**).

Our core genome based phylogeny analysis clearly indicated that all the 42 genomes have been distributed in three compact clades and the tree is distinct from the pan-genome based phylogenetic tree (**Figure 5**). Since all the strains of each clade are very close to each other and most are diverged from a single LCA, it reflects that all protein sequences of 316 genes are highly conserved and sequence similarity percentage is very narrow from 69.03 to 100 (**Supplementary Table S5**).

### Genome of *L. crispatus and L. gasseri* Enriched With Secretory Proteins With Potential Gene Regulatory and Antimicrobial Functions

Analysis of the representative genomes of *L. crispatus* and *L. gasseri* indicate presence of several secretory transcriptional regulators and several antimicrobial peptides correlated with less diverse microbial composition and also anti-inflammatory condition in the vagina. We have identified 36 and 19 secretory signal peptides containing DNA binding motif in the genome of *L. crispatus* and *L*. *gasseri* strains, respectively (**Supplementary Table S6**). The site of localization of these secretory transcriptional regulators have been predicted either the nucleus or mitochondria of host cells. This subcellular localization information gives an important clue to the transcriptional regulatory functions of the secretory proteins. Although localization signals in mRNA appear to play some role (Gonsalvez et al., 2005), but the key determinant of protein localization is the peptide linked with the N-terminal end of the protein molecules. The present finding indicates that these secretory proteins may come out from the *Lactobacillus* species and enter into host cell and act as transcription regulator. Consequentially the host genome may change the expression level of pro- or anti-inflammatory proteins or antimicrobial peptides to modulate the microbial growth and reduce the invasion of pathogenic bacteria.

## Discussion

The atypical composition of the vaginal microbiome in genetically predisposed women is a potential environmental risk factor for PTB, which leads to an ascending migration of specific bacterial taxa from the vaginal milieu to amniotic membrane and amniotic fluid. It possibly induces an aberrant immune response and activation of several matrix associated enzymes leading to activation of preterm labor (Alamrani et al., 2017). There has been a recent surge in the information on the role of vaginal microbiome in PTB; In general PTB has been characterized by increased microbial diversity, decline in anti-inflammatory molecules and rise in pro-inflammatory bacteria in the vaginal milieu (Parris et al., 2020). Vaginal microbiota of asymptomatic, otherwise healthy women is mostly dominated by different species of *Lactobacillus* (Ravel et al., 2011; Mehta et al., 2020; Ma et al., 2020). In the present study, *L. crispatus* and *L. gasseri* have been commonly found to be associated with term birth outcome in in accordance with the other previous reports (Kindinger et al., 2017; Stafford et al., 2017). Dominance of *Lactobacillus* species in the vaginal microbiome of white American and Asian women is more common than the Black American and Hispanic women (Ravel et al., 2011). *Lactobacillus* species protect the vaginal milieu from colonization and growth of exogenous and potentially pathogenic bacterial taxa by producing lactic acid, hydrogen peroxide (H_2_O_2_), maintaining acidic pH of the niche and secreting ribosomally encoded antimicrobial peptides (Amabebe and Anumba, 2018). In addition, lactic acid producing bacteria induce host innate immune system while sensing presence of non-indigenous Gram-negative bacteria in the vaginal milieu (Witkin et al., 2011). *In vitro* colonization studies using vaginal epithelial cell line with *L. crispatus* and other native microbiota demonstrated distinct immunity of epithelial monolayer in a species-specific manner (Lai et al., 2009). In the present study, we characterized the vaginal microbiota of 115 HVS samples collected longitudinally from the 38 pregnant women who were enrolled in the GARBH-Ini pregnancy cohort. The data obtained from our study population indicates that like reports from other populations vaginal pH of the reproductive aged Indian women is also acidic in nature (pH 5.0 ± 0.55). During pregnancy there is a rise in level of progesterone and estrogens, along with some immunological changes which in turn increase the glycogen content of the vaginal epithelial cells and modulate the composition of the vaginal microbiome to a more stable state; this reduces the richness and community diversity by promoting the growth of *Lactobacillus* sp. (Marchesi and Ravel, 2015). Dominance of *Lactobacillus* provides a greater resistance and protective role against genital tract infections (Marchesi and Ravel, 2015). Our high- throughput sequencing reads covering V3-V4 region of 16S rRNA genes allowed us to accurately determine the composition, diversity and dynamics of the vagina microbial ecosystem in asymptomatic Indian women who delivered preterm.

The dominance of non-*Lactobacillus* species in the vaginal milieu has previously been reported as potential risk factor for PTB (Moreno and Franasiak, 2017; Drew et al., 2018). In our study we found that the taxa, which have previously been found to be associated with Bacterial Vaginosis (BV) (Srinivasan et al., 2012) were significantly higher in preterm delivering women compared to term delivering women. When we compared the alpha diversity indices such as Shannon and Chao1 in all the three trimesters of pregnancy between term and preterm samples and observed that the diversity indices are slightly higher in preterm samples but the differences are not statistically significant (**Figure 1**). A total of 217 and 244 bacterial genera were identified in the term and preterm samples, respectively. We have previously reported that the most dominant bacterial taxa in the reproductive age Indian women are *Lactobacillus* (Mehta et al., 2020). Dominance of *Lactobacillus* in the vaginal microbial ecosystem of reproductive age healthy women has also been reported from several other countries and our findings are consistent (Zhou et al., 2007; Drell et al., 2013).

In the present study, we also observed that *L. crispatus*, *L. iners, L. gasseri*, *L*. *jensenii* and *L. delbrueckii* are the dominant species both in term and preterm samples. However, the relative abundance of *L. iners* is high in preterm samples, while the relative abundance of *L. crispatus* and *L. gasseri* is high in term samples. Similar abundance profile of *L. crispatus*, *L. gasseri* and *L. iners* in term and preterm samples have also been reported in other cohorts studying the role of microbiome in birth outcomes (Tabatabaei et al., 2019).

When we analyzed the relative abundance of other non-*Lactobacillus* bacterial taxa we observed higher abundance of a mixture of facultative anaerobic bacteria such as *Gardnerella*, *Sneathia* and *Megasphaera* in the vaginal milieu. At the species level resolution of 16S rRNA gene sequence reads revealed that the relative abundance of *Sneathia sanguinegens* and *Gardnerella vaginalis* is significantly higher in women delivering preterm compared to the women having term delivery. No claim is made that the significant differences noted in this study are final; this should be viewed as tentative findings that require validation using independent cohorts of mothers. Preterm Premature Rupture of the fetal Membrane (PPROM) is associated with *Sneathia* (Brown et al., 2018). It was previously shown that *G. vaginalis* acts as a preterm signature in European ancestry (Callahan et al., 2017). Although, similar reports from other cohorts are available (Romero et al., 2014; Seo et al., 2017; Tabatabaei et al., 2019), the genomic content of these abundant bacterial species from the same cohorts have not been explored. For a better and more profound understanding, how the presence or absence of a bacterial species effect the composition of a microbial ecosystem or host physiology, it is important to decode their genome and identify the pertinent functions. In the present study, for functional insights that may link with the birth outcomes we isolated several strains of the three most important *Lactobacillus* species i.e. *L. crispatus*, *L. gasseri* and *L. iners.* A comparative genome analysis of *L. crispatus, L. gasseri* and *L. iners* has revealed that *L. crispatus* isolate genomes are enriched with additional genes related to lactose, galactose, sucrose and fructose fermentation that leads to lactic acid production. Further analysis reveal that different genes linked with defense mechanisms are part of the shell genome and each species has some unique genes to protect themselves in the complex microbial ecosystem. A similar study also supports our findings that a set of unique transposable elements, multidrug resistance protein (MdtG), sensor histidine kinase (RcsC) and phosphate-binding protein (PstS) are prevalent in the genome of *L. crispatus*. Such functions help *L. crispatus* to compete with exogenous microbiota, exclude them from the colonization in the vaginal milieu and keep the environment less diverse and protected from enrichment of pro-inflammatory molecules, mostly produced by the non-indigenous vaginal microbiota. In addition, the genome of *L. iners* harbors thiol-activated cytolysin (TACY). It is an important group of bacterial toxins, of which streptolysin O (SLO) is the prototype of TACY. They are involved in the pathogenesis of a number of Gram-positive species. TACY are pore-forming toxins, their major pathogenic effects may be more delicate than simple lysis of host cells, and may include interference with immune cell function and cytokine induction. This cytolysin was not detected in the genome of *L. crispatus* and *L. gasseri* analyzed in the present study.

Further analysis of the pan- and core genomes of *L. crispatus, L. gasseri* and *L. iners* revealed that each species have distinct genomic contents and they are clearly diverged from each other. We identified 316 core genes in the genomes of all three *Lactobacillus* species (**Supplementary Table S7**). Similar core gene contents among different *Lactobacillus* species have also been reported by the other groups (Inglin et al., 2018; Evanovich et al., 2019; Putonti et al., 2020). The pool of core and soft-core subsets represent highly conserved gene cluster, as these clusters are present in ≥95% of the 42 genomes. The soft-core subset has additional important in comparative genomic analysis as it allows inclusion of draft genomes in which some genes may not be present (Nelson and Stegen, 2015). Therefore functional annotation of core and soft-core gene clusters can provide information about fundamental and essential cellular processes of the *Lactobacillus* genus (Carlos Guimaraes et al., 2015). There are eight classes in which either core or soft-core gene clusters are predominantly present. They are: Nucleotide transport and metabolism (class: F), Coenzyme transport and metabolism (class: H), Translation, ribosomal structure and biogenesis (class: J), Replication, recombination and repair (class: L), Cell wall/membrane/envelope biogenesis (class: M), Cell motility (class: N), Posttranslational modification, protein turnover, chaperones (class: O), Intracellular trafficking, secretion, and vesicular transport (class: U). These classes represent core functions of a prokaryotic organism. Therefore, pool of core and soft-core subsets may call as dispensable genome.

Similarly, there are ten functional classes in which pool of shell and cloud subsets (either shell or cloud or both subset) of pan-genome are predominantly present. They are following: Transcription (class: K), Energy production and conversion (class: C), Amino acid transport and metabolism (class: E), Carbohydrate transport and metabolism (class: G), Lipid transport and metabolism (class: I), Inorganic ion transport and metabolism (class: P), General function prediction only (class: R), Function unknown (class: S), Signal transduction mechanisms (class: T) and Defence mechanisms (class: V). These functions are part of the shell and cloud genomes and they perform important functions and help *Lactobacillus* to compete with other microorganisms living in the same vaginal milieu. Another study shows that shell and cloud genomes contain virulence factors, antibiotic resistance genes and different metabolic enzymes important for the survival of the microorganism in specific environments(Mira et al., 2010; Read and Ussery, 2006). Therefore, gene clusters of the pool of shell and cloud subsets may be called as flexible/accessory genome as they present in ≤95% of the 42 genomes. Analysis of accessory genome may reveal both the evolutionary history of a sub lineage or isolates and their adaptability in different environment (Nelson and Stegen, 2015). These two subsets (i.e. shell and cloud) of pan-genome are thought to have different rates of gene acquisition and deletion through horizontal gene transfer (Collins and Higgs, 2012). It is believed that gene gained and lost slowly happen in shell, whereas comparatively fast in cloud (Collins and Higgs, 2012). Therefore, it is believed that the unique gene cluster comes into cloud subset.

However, pangenome based analysis reveals that *Lactobacillus* species isolated in the present study have several unique genes acquired through horizontal gene transfer (**Supplementary Table S7**). It was observed that the *L. crispatus* clade is more scattered than other two clades. The different branch length of each of the *Lactobacillus* species indicates that the ancestors for these species were also reasonably different. Different functions that are unique to *L. crispatus* genome have potential antimicrobial activity against different opportunistic pathogens like *A. baumanii* and *K. pneumoneae*. Several Gram-negative bacteria associated with microbial dysbiosis in the vaginal milieu are correlated with production of proinflammatory cytokines and induction of labor. Thus the antimicrobial peptides produced by the *L. crispatus* can reduce the abundance of pro-inflammatory molecules in the vaginal milieu by reducing the colonization and growth of pathogenic bacterial taxa. Our findings indicate that *L. crispatus*, *L. gasseri*, *L. iners. S. sanguinigens* and *G. vaginalis* genome specific signature could be used as the microbial genome signature for predicting birth outcomes. However, this study has certain limitations. Our findings indicate that there is a correlation between some Lactobacilli species and term delivery, but this alone does not explain the protective effect. Sample size of the present study is also not adequate and need validation with larger sample size for a definitive conclusion.

## Conclusion

The composition and diversity of vaginal microbiota widely vary across populations. Specific microbial taxa contribute substantially in determining term or PTB outcomes. We observed that the higher abundance of *L. crispatus* and *L. gasseri* are associated with TB while the increased abundance of *S. sanguinigens* and *G. vaginalis* are linked with PTB. Prevalence of *L. iners* is also high in mothers having PTB. The genome of *L. crispatus* and *L. gasseri* are enriched with horizontally acquired genetic elements and peptides potentially linked with antimicrobial functions. Such bacterial taxa reduce the microbial diversity in the vaginal milieu and also the microbial origin inflammation inducing antigens. The microbial taxa and their genomic signatures linked with birth outcomes reported in the present study need to be validated with other population. In addition, the present study has limited sample size. Multicenter studies with larger sample size will help to understand whether the observed microbial taxa and their genomic contents associated with TB and PTB births have any link to a specific race or ethnicity.

## Data Availability Statement

The original contributions presented in the study are publicly available. This data can be found here: European Nucleotide Archive (ENA) with identifier PRJEB43005. Genome Accession: JAGSXW000000000, JAGSXV000000000, JAGSXU000000000, JAGSXT000000000, JAGSXS000000000, JAGSXR000000000, JAGSXQ000000000, JAGSXP000000000, JAGSXO000000000, JAGSXN000000000.

## Ethics Statement

The studies involving human participants were reviewed and approved by Translational Health Science and Technology Institute Human Ethics Committee. The patients/participants provided their written informed consent to participate in this study. [Ref.# THS 1.8.1/(30) dated 11th Feb 2015].

## Collaborative Authors

**MEMBERS OF GARBH–Ini** (in alphabetical order of surnames): Translational Health Science and Technology Institute, NCR Biotech Cluster, Faridabad, India-Coordinating Institute (Shinjini Bhatnagar (PI), Bhabatosh Das, Vineeta Bal, Bapu Koundinya Desiraju, Pallavi Kshetrapal, Sumit Misra, Uma Chandra Mouli Natchu, Satyajit Rath, Kanika Sachdeva, Dharmendra Sharma, Amanpreet Singh, Shailaja Sopory, Ramachandran Thiruvengadam, Nitya Wadhwa); National Institute of Biomedical Genomics, Kalyani, West Bengal, India (Arindam Maitra, Partha P Majumder (Co-PI) Souvik Mukherjee); Regional Centre for Biotechnology, NCR Biotech Cluster, Faridabad, India (Tushar K Maiti); Clinical Development Services Agency, Translational Health Science and Technology Institute, NCR-Biotech Cluster, Faridabad, India (Monika Bahl, Shubra Bansal); Gurugram Civil Hospital, Haryana, India (Umesh Mehta, Sunita Sharma, Brahmdeep Sindhu); Safdarjung Hospital, New Delhi, India (Sugandha Arya, Rekha Bharti, Harish Chellani, Pratima Mittal); Maulana Azad Medical College, New Delhi, India (Anju Garg, Siddharth Ramji), The Ultrasound Lab, Defence Colony, New Delhi, India (Ashok Khurana); Hamdard Institute of Medical Sciences and Research, Jamia Hamdard University, New Delhi, India (Reva Tripathi); All India Institute of Medical Sciences, New Delhi, India (Yashdeep Gupta, Smriti Hari, Nikhil Tandon); Government of Haryana, India (Rakesh Gupta); International Centre For Genetic Engineering and Biotechnology, New Delhi, India (Dinakar M Salunke Co-PI); G Balakrish Nair (Rajiv Gandhi Centre for Biotechnology, Trivandrum)

## Author Contributions 

BD, SB, and GN conceived the idea and designed the experiments. Members of GARBH-Ini conducted the clinical study, collected HVS samples and relevant clinical information. SK, NK, DT, AK, MS, OM, BD, GARBH-Ini study group, SM, and BD performed experiments. BD, SM, SB, PK, RT, NW contributed reagents. BD, SM, SK, NK, DT, and MS performed data analysis. BD and SM wrote the manuscript. SB, NW, TR, and PK edited the manuscript. All authors contributed to the article and approved the submitted version.

## Funding

The work was funded by the Department of Biotechnology, Govt. of India (No.BT/PR9983/MED/97/194/2013), Translational Research Program (TRP) of the THSTI and, for some components of the biorepository, by the Grand Challenges India–All Children Thriving Program, Biotechnology Industry Research Assistance Council (grant BIRAC/GCI/0114/03/14-ACT). 

## Conflict of Interest

The authors declare that the research was conducted in the absence of any commercial or financial relationships that could be construed as a potential conflict of interest.

## Supplementary Material

Supplementary Figure S1 Rarefaction curve for alpha diversity. **(A)** Chao1 and **(B)** Shannon diversity index.
